# Variability in the inorganic composition of colored acrylonitrile–butadiene–styrene and polylactic acid filaments used in 3D printing

**DOI:** 10.1007/s42452-022-05221-7

**Published:** 2022-12-08

**Authors:** Derek M. Peloquin, Logan N. Rand, Eric J. Baumann, Ali Gitipour, Joanna Matheson, Todd P. Luxton

**Affiliations:** 1 Center for Environmental Research and Emergency Response, Office of Research and Development, U.S. Environmental Protection Agency, 5995 Center Hill Ave, Cincinnati, OH 45224, USA.; 2 Present Address: Forensic Chemistry Center, Office of Regulatory Affairs, U.S. Food and Drug Administration, Cincinnati, OH 45237, USA.; 3 Present Address: Center for Environmental Measurements and Modeling, Office of Research and Development, U.S. Environmental Protection Agency, Research Triangle Park, NC, USA.; 4 Office of Hazard Identification and Reduction, U.S. Consumer Product Safety Commission, Bethesda, MD, USA.

**Keywords:** Fused filament fabrication, Digestion methods, Inorganic composition, Metal speciation, Polymer inorganic composition, 3D printing, Additive manufacturing

## Abstract

Fused filament fabrication is a 3D printing technique that has gained widespread use from homes to schools to workplaces. Thermoplastic filaments, such as acrylonitrile–butadiene–styrene (ABS) and polylactic acid (PLA), are extruded at temperatures near their respective glass transition temperature or melting point, respectively. Little has been reported on the inorganic elemental composition and concentrations present in these materials or the methods available for extracting that information. Because inorganic constituents may be included in the aerosolized particulates emitted during the printing process, identifying elements that could be present and at what specific concentrations is critical. The objective of the current research is to determine the range of metals present in thermoplastic filaments along with their relative abundance and chemical speciation as a function of polymer type, manufacturer, and color. A variety of filaments from select manufacturers were digested using a range of techniques to determine the optimal conditions for metal extraction from ABS and PLA polymers. The extraction potential for each method was quantified using by ICP-MS analysis. When possible, further characterization of the chemical composition of the filaments was investigated using X-ray Absorption spectroscopy to determine chemical speciation of the metal. Optimal digestion conditions were established using a high temperature, high pressure microwave-assisted acid digestion method to produce the most complete and repeatable extraction results. The composition and abundance of metals in the filaments varied greatly as a function of polymer, manufacturer, and color. Potential elements of concern present in the filaments at elevated concentration included that could pose a respiratory risk included Si, Al, Ti, Cu, Zn, and Sn. XAS analysis revealed a mixture of metal oxides, mineral, and organometallic compounds were present in the filaments that were being used to increase opaqueness impart color (dyes), polymeric catalysts, and flame retardants. This work shows that a variety of metals are present in the starting materials used for 3D printing and depending on their partitioning into 3D printed products and byproducts as well as the exposure route, may pose a health risk which merits further investigation.

## 1 Introduction

Fused filament fabrication (FFF) is a 3D printing technology that is part of a multi-billion-dollar industry, with an ever-increasing presence in homes, public spaces (such as libraries), and classrooms [1–3]. The technology is revolutionizing the manufacturing industry and making it possible to operate “mini” manufacturing enterprises from the home [4, 5]. In the FFF printing process, a thermoplastic polymer filament is extruded through a nozzle near the melting temperature to produce 3-dimensional objects through additive layers. Filaments are sold by a wide array of manufacturers in a variety of polymers, colors, and additives. Additives may include, but are not limited to, metals (copper, bronze, stainless steel), nanomaterials, and flame retardants [6–8]. Based on a survey of online product suppliers and previous research, acrylonitrile–butadiene–styrene (ABS) and polylactic acid (PLA) are the most commonly used thermoplastics for FFF [9]. Little information or research is publicly available on the inorganic composition of the thermoplastics and this presents an additional knowledge gap in the toxicological hazards of the household use and products of 3D printing.

Recent attention regarding the environmental fate of micro and nanoplastics has led to new efforts to determine their inorganic constituents using a variety of analytical methods. Srinidhi et al. [10] published a comprehensive review of the effects of micro and nanoplastics on biological organisms, including polymer types, additives, and human versus animal impacts. Listed potential inorganic plastic additives and their purpose include Pb, Cu, Pd, Hg, Cd, and zeolites as residual catalysts, silica, zeolites, and talc as antiblocking agents, phosphites as antioxidants, Cd and Zn salts as stabilizers, Molybdenum compounds as lubricants, clay, silica, and carbonate minerals as fillers, TiO_2_ for color pigmentation, and silicones as cross-linking compounds. Because most additives are not chemically bonded to the polymer, they have greater potential for release. Hahladkis et al. present a robust overview of plastic additives and consequential life cycle considerations and previous studies on thermoplastic polymers have demonstrated that it is not uncommon to have μg/g concentrations of metals present [11–15]. Skrzydlewska and Balcerzak [11] performed acid-assisted microwave digestions on low density polyethylene (LDPE), polyethylene-polypropylene (PE-PP), and high density polyethylene (HDPE) non-food packaging materials. Inductively coupled plasma (ICP) time-of-flight mass spectrometry analysis revealed that the two LDPE samples had high concentrations of Cu (112 and 49.3 μg/g), the PE-PP sample had high concentrations of Zn (449 μg/g) and Pb (160 μg/g), and that the older HDPE sample had high concentrations of Pb (1222 μg/g), Cr (219 μg/g), Cd (113 μg/g), Ba (57.4 μg/g), and Sb (44.1 μg/g), while the newer sample was below 1 μg/g for all of those elements, which they equated to a change in permissible pigment materials. Turner [13] collected marine litter in the form of foams, ropes, and plastics from five beaches in the United Kingdom and found 14 elements detected across 279 pieces of plastic, identified primarily as PE and PP. The elements with the highest median values were Ba (1690 μg/g over 39 samples), Cd (1270 μg/g over 11 samples), Cl (672 μg/g over 225 samples), and Sb (262 μg/g over 18 samples), though nearly all median concentrations were > 20 μg/g due to detection and measurement by field-portable X-ray fluorescence spectrometry. ABS, PP, polycarbonate, and high impact polystyrene made up over 65% of the waste electrical and electronic equipment plastic that was tested by Stenvall et al. [12] from two different recycling plants. Leaching of the plastics with nitric or citric acid followed by analysis using ICP optical emission spectroscopy showed most of the toxic heavy metals were undetectable but that some samples concentrations of Pb were greater than 100 μg/g, along with Al, Ca, Fe, and Zn. Nham of Agilent Technologies performed a comparison of three different digestion methods for PE and ABS reference materials: European Standard EN 1122, EPA Method 3051A, and an HNO_3_-H_2_O_2_ digestion, followed by measurement of Cd, Cr, Hg, and Pb [16]. Recovery of Cd proved to be acceptable across all three methods, but while Cr and Pb had acceptable recoveries with the other two digestions, Method 3051A was the only method suitable for Hg found in the PE material.

Recent research has indicated a need for greater understanding the chemical composition of 3D printing aerosol emissions and their health hazards [17–27]. Steinle [16] measured the ultrafine aerosols (UFAs) and volatile organic compounds (VOCs) from printing with ABS and PLA filaments in two different workplace environments and found that the emissions corresponded primarily to volatile droplets, which were found as amorphous particles collected on TEM grids. The handful of metal-containing solid particles that were identified were attributed to either printer, filament, or environmental contamination and after seven months of use, the printer was producing more UFAs than when initially purchased. Zontek et al. [21] instead found 11 elements in the ultrafine particles collected from printing with ABS and PLA filaments, and their results agreed with Steinle’s characterization of the aerosol emissions. Carbon and oxygen were the most abundant, indicating a likely combustion process occurring during printing, but elements such as Al, Cu, and Si were also identified. When comparing ABS, PLA, and PC filaments containing carbon nanotubes (CNTs) to their base versions, Stefaniak et al. showed that CNTs completely partitioned into both large particulates in the aerosol fraction and into the printed products [23]. Although no elemental analysis was performed in this study, their results show the possibility of larger non-volatile species being found in the aerosol emissions of printing processes.

Stefaniak et al. measured the inorganic composition in the bulk feedstock resins, aerosolized particles, and printed objects related to vat polymerization 3D printing processes [28]. They measured 5 to 7 elements in the bulk resins, 5 to 9 elements in the aerosolized particles, and a minimum of 18 elements in the printed objects; the elements detected in the particles only corresponded with 1 to 3 elements in the bulk resins, and the printed objects had elements at higher concentrations than those detected in the resins. In the only study to our knowledge in which the inorganic concentrations were measured from FFF filaments, Yi et al. [1] looked at the materials that would be used in 3D printing toy pens and printers. They found that Al, Fe, and Zn were the most abundant metals measured in the 3 PLA filaments and 1 ABS filament, though at ng/g levels. Using scanning electron microscopy (SEM) coupled to energy-dispersive X-ray spectroscopy, they found particles containing Fe in emissions from both pens and printer. ICP mass spectrometry (ICP-MS) analysis of filters collected during printing showed the highest yields of Fe (3168 ng/g for a printer with translucent blue filament, 439.5 ng/g for one printing pen with clear orange filament, and 127.0 ng/g for a second pen with clear yellow filament) and Al (372.4 ng/g for the second pen with clear yellow filament).

The goal of the study presented here is to fully investigate the inorganic components of FFF filaments, which required development of a robust analytical method capable of quantifying elements across the periodic table. Based on previous literature characterizing metals in plastic, digestion followed by metal quantification by ICP-MS was the chosen approach with four different digestion methods compared for suitability. Following this procedure, a baseline dataset was determined for a cross-section of different types of plastics, manufacturers, colors, and additives commercially available for desktop 3D printers. This provides an estimate of the range and variation in the composition and concentration of the elements present in FFF thermoplastic starting materials. Speciation by x-ray absorption spectroscopy was additionally carried out for selected filaments. The composition results determined here are useful to guide further research investigating the potential exposure hazards of commercially available FFF printing. Due to the rapidly expanding availability and applications of this industry, continued assessment of the inorganic chemical risks associated with this technology is warranted.

## 2 Material and methods

Fourteen filaments consisting of two types of plastics (ABS and PLA) in different colors (black, blue, and green, including some variable hues) were obtained from three manufacturers at different price points—color images of filaments available in supporting information (Fig. S1). To avoid identifying specific companies the manufactures of different filaments are labeled Manufacturer 1, 2, and 3. ABS and PLA filaments (black, green, and blue for both polymers) were purchased from Manufacturer 1 at a price point of $20 kg^−1^. Only ABS filaments in blue and green were purchased from Manufacturer 2 at a $30 kg^−1^ price point. Finally, blue, green, and black ABS and PLA filaments were purchased from Manufacturer 3 at a $40 kg^−1^ price point. All filaments used in the current study were 1.75 mm in diameter and had a nominal density of 1.24 and 1.05 g cm^−3^ for PLA and ABS, respectively. The manufacture recommended printing temperatures for PLA were the same for Manufacturer 1 and 3 (205 ± 15 °C). The recommended print temperatures for ABS were 220 ± 5 °C for Manufacturer 1 and 2 and 235 ± 5 °C for Manufacturer 3.

### 2.1 Digestion methods

Each filament digestion sample was divided into equal-sized pieces weighing a total of 0.1 ± 0.01 g. Digestions were performed in three to five replicates using four methods: EPA Method 3051A microwave-assisted acid digestion (hereafter labeled as M); high temperature-modified EPA Method 3051A (labeled as HT); high pressure, high temperature-modified EPA Method 3051A (labeled as HPHT); and EPA Method 3050B, hot block acid digestion (labeled as HB).

For the M method, samples were transferred into perfluoroalkoxy (PFA) MARSXpress microwave digestion vessels (CEM Corporation, Matthews, NC) with 9 mL of concentrated nitric acid ( HNO_3_, TraceMetal^™^ grade, Fisher Scientific, Waltham, MA) and 3 mL of concentrated hydrochloric acid (HCl, TraceMetal^™^ grade, Fisher Scientific) added. Sample vessels were heated to 175 ± 5 °C with a 5:30-min ramp time and 4:30-min hold time using the MARS 6 microwave digestion unit (CEM Corporation).

Samples digested using the HT method were also prepared in PFA MARSXpress vessels with 9 mL of HNO_3_ and 3 mL of HCl but were heated to 200 ± 5 °C with a 20-min ramp time and 20-min hold time.

The HPHT method was performed using TFM iPrep digestion vessels (CEM Corporation) with 9 mL of HNO_3_ and 3 mL of HCl that were heated to 200 ± 5 °C with a 20-min ramp time and 20-min hold time. iPrep vessels have a maximum pressure of 1500 psi (103 bar) versus 500 psi (35 bar) for the MARSXpress vessels and withstood all digestions without any venting of contents.

For the HB method, samples were placed in 50 mL PFA digestion tubes (Questron Technologies Corporation, Mississaugua, ON) and heated to 95 ± 5 °C with a total of 25 mL of HNO_3_, 8 mL of 30% nonstabilized hydrogen peroxide (H_2_O_2_), and 5 mL of HCl added in separate portions over ~ 8 h using the QBlock PTFE-coated digestion block (Questron Technologies Corporation).

After digestion, all supernatants were collected and diluted to 50 mL with ultrapure water from a Super-Q water purification system (MilliporeSigma, Burlington, MA) and stored at 4 ± 2 °C in 50 mL PP centrifuge tubes (Fisher Scientific).

### 2.2 Inductively coupled plasma mass spectrometry (ICP-MS)

All digested samples were analyzed for total metals concentrations using the Agilent 7900 ICP-MS (Agilent Technologies, Santa Clara, CA) with or without helium collision mode depending on the element in accordance with EPA Method 6020B. The instrument was tuned using a 10-ppb solution of Ce, Co, Li, Tl, and Y in 5% nitric acid (Inorganic Ventures, Christiansburg, VA) and a 1-ppm solution of Li, Sc, Ge, Y, In, Tb, and Bi in 5% nitric acid (Inorganic Ventures) was used as an internal standard. The glassware used consisted of 0.4 mL/min flow concentric glass MicroMist^™^ nebulizer, quartz Scott-style spray chamber, straight quartz connector, and quartz torch with 2.5 mm injector (Glass Expansion, Pocasset, MA). Instrument settings consisted of 1500 W RF power, 15.00 L/min plasma gas flow rate, 1.1 L/min carrier gas flow rate, 0.9 L/min auxiliary gas flow rate, and 2 °C spray chamber temperature. Method detection limits (MDLs) and method reporting limits (MRLs) were determined separately for each digestion method according to 40 CFR Appendix B to Part 136 and are provided in Table S1 in the supporting information. In the results for all samples, the values from individual replicates were either presented as measured when greater than the MDL or as one-half of the MDL when less than the MDL, as long as the average of the replicates was greater than the MDL.

### 2.3 X-ray absorption spectroscopy (XAS)

All filament samples analyzed by XAS were prepared by abrading the filament with 150 grit sanding discs (Diablo Tools, High Point, NC) to yield homogeneous plastic powders. Powders were combined with polyvinylpyrrolidone (PVP), pressed into 1.3 mm pellets using a manual pellet press, then sealed in Kapton polyimide tape. Pellets were measured for Fe K-edge XAS at the Materials Research Collaborative Access Team (MRCAT) 10-ID beamline at the Advanced Photon Source (APS) operated by the U.S. Department of Energy (DOE) at Argonne National Laboratory (Lemont, IL) [29] and for Ti, Cu, Zn, and Sn K-edge XAS at the MRCAT 10-BM beamline [30]. For Fe XAS, the energy of the incident X-rays was scanned using an Si(111) monochromator consisting of a cryogenically-cooled first crystal and a 250-mm long second crystal and with a SiO_2_ harmonic rejection mirror. Incident beam energy was calibrated to the first derivative inflection point of the absorption edge (7112 eV) of an Fe foil reference standard. Spectra were collected in transmission mode, using an ion chamber, and fluorescence mode, using a Lytle-type fluorescence detector. For Ti, Cu, Zn, and Sn XAS, the energy of the incident X-rays was scanned using an Si(111) monochromator consisting of a water-cooled first crystal and a 50-mm long second crystal. Incident beam energies were calibrated to the first derivative inflection points of the absorption edges (4966 eV for Ti, 8979 eV for Cu, 9659 eV for Zn, and 29,200 eV for Sn) of corresponding metal foil reference standards. Spectra were collected in transmission mode, using an aluminum spectroscopy ion chamber, and in fluorescence mode, using a 4-element Si drift diode detector. Merging of the measured spectra was first performed using LARCH [31], followed by spectral alignment, energy calibration, background removal, and normalization using ATHENA [32]. Linear combination fitting (LCF) was performed with appropriate standards on the normalized spectra of the samples using ATHENA with a fitting range of − 30 to 100 eV relative to the absorption edge.

### 2.4 Statistical analysis

All plots and statistical analysis were prepared or performed using OriginPro 2020 from Origin Lab Corporation (Northhampton, MA). A one-way ANOVA followed by pairwise comparison of means between treatments via a Tukey test (*α* = 0.05) was used first to determine statistical differences in the quantity of metals extracted by each digestion method, then to examine differences in concentrations between filament manufacturer and color.

## 3 Results and discussion

### 3.1 Comparison of the digestion methods

Prior to comparing the elemental composition between different materials, manufacturers, and colors, it was necessary to first evaluate appropriate methods for maximum extraction of elements from the printer filaments. While previous studies have used several different methods to extract inorganic constituents from different plastic polymers, to our knowledge there has been no evaluation of these methods when applied to the thermoplastic materials used in 3D printing. As a starting point for a systematic approach, the element detectability as a function of digestion method was first determined. This was accomplished by comparing the average concentration of the digestate (3–5 replicates for each filament and method) against the instrument MDLs and MRLs (provided in Table S1 in the supporting information). Because the elemental composition and concentration in each of the filaments varied widely, the robustness of the different digestion methods was evaluated separately for each specific filament type (ABS, PLA, and metal additive filaments). Statistical differences in the quantity of elements extracted from each filament as a function of the digestion method type were determined via ANOVA and Tukey Means Comparison analysis. Elements were included based on three criteria: (1) the element must be present at quantifiable concentrations (SI Table S1), (2) the element is present in at least 50% of the filaments digested using a given method, and (3) the element must meet criteria 1 and 2 for at least two different digestion methods. Based on the selecation criteria Mg, Al, Si, P, K, Ca, Ti, and Cu were used in the comparison of digestion methods for ABS filaments and Ca, Ti, V, Cu, and Sn were included for the PLA filaments. (SI Tables S2, S3).

**Fig. 1:**
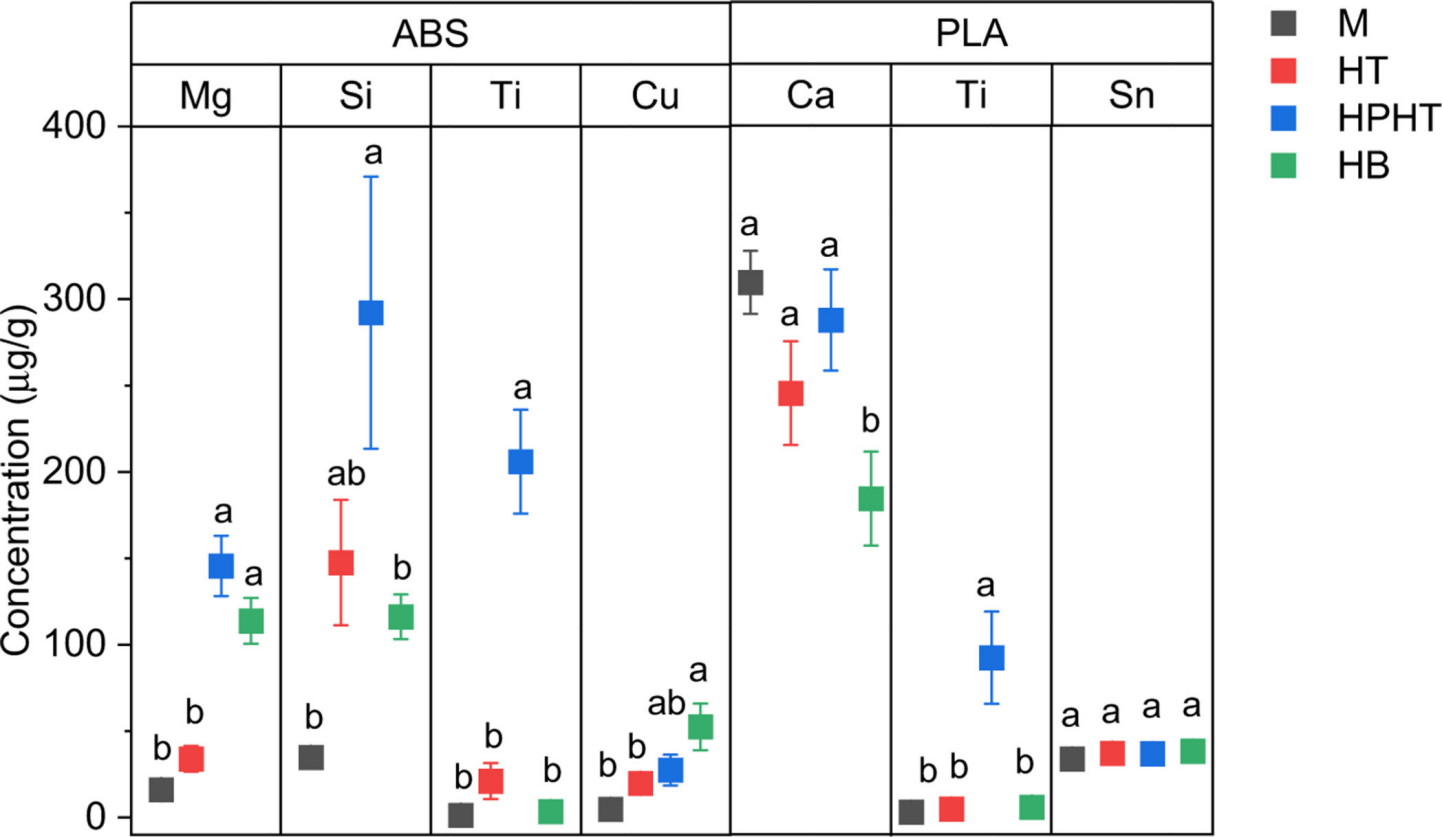
Concentration of elements recovered from ABS and PLA plastics as a function of the digestion method. Error bars are equal to the standard error calculated in the ANOVA. Lower case letters correspond to the results from the Tukey Means pair wise comparison. A difference in letters indicates a statistical difference in the mean concentration of an elements extracted at an *α* = 0.05

Results from the ANOVA and Tukey Means comparison are presented in Table 1, with the corresponding mean values and standard error presented in Table S4 and S5 in the SI and in Fig. 1 for selected elements. The specific digestion method used resulted in significant differences in the quantity extracted for 10 of the 21 elements identified in Table 1. Element extractability of the ABS filaments were more impacted by the specific digestion method compared to the PLA polymer. With respect to the PLA filaments, the specific digestion method used resulted in statistical differences for only two elements (Table 1). Overall, the HPHT digestion method extracted the greatest quantity of elements for 11 of the 21 elements. In every case, the HPHT digestion method extracted the greatest quantity of elements or there was no statistical difference between the HPHT method and the method that did extract the largest elemental concentration.

Max refers to the digestion method that extracted the maximum elemental concentration for a specific polymer and element. Statistically significant differences between digestion methods are denoted with lowercase letters, such that “a” and “b” results are different from each other, “a” results are not significantly different from each other, “b” results are not significantly different from each other, and “ab” results are not significantly different from “a” or “b” results. The p-value presented is from a single factor ANOVA where digestion method was the factor.

A subset of the results from the ANOVA and Tukey Means comparison analysis for ABS and PLA filaments are presented in Fig. 1. In some instances where digestion methods were significantly different, the difference between the concentration of an element extracted could differ by over an order of magnitude (Fig. 1), with a particularly noticeable increase in the amount of Ti and Si extracted from ABS and PLA filaments by the HPHT method. It is apparent that ABS filaments and filaments containing Ti particles required an aggressive digestion method for maximum extraction and recovery (Fig. 1; Table S4). The elevated concentration of both Ti and Si extracted in the HPHT digestion method that would have been missed with the other methods is critical for a complete characterization and risk assessment of filaments. Both Ti and Si are frequently used in polymer fabrication and expected to be commonly present in many plastic feedstock materials. Ti is often present as anatase (TiO_2_) and used for a variety of reasons, namely UV-resistance and opacity [33], while Si may be related to either a siloxane based flame retardant or silica (SiO_2_) [34–36].

Out of the four methods, the HPHT method requires digestion vials that are more specialized in order to withstand the higher temperature and pressure. If the HPHT equipment is unavailable, the HT method is recommended due to its elevated temperature and extraction time compared to the M method. This increased temperature does lead to greater instances of venting during the digestion which may result in analyte loss, but this can be minimized by initially allowing samples to digest in the acid at room temperature for 12 h. It should be noted that performing the HB method for PLA filaments or others that might have Sn included, only non-stabilized hydrogen peroxide should be used for digestion, since commercially stabilized hydrogen peroxide may include Sn compounds and can easily raise the MDLs and MRLs to unusable levels [37]. For the remainder of the study presented here, the dataset obtained by the HPHT method are used to examine filament composition by material, manufacturer, and color.

### 3.2 Comparison of the plastics and manufacturers

Of the 28 elements measured, 5 elements showed consistent differences between PLA and ABS filaments: Al, K, Mg, Si, and Sn. Table 2 presents the average concentration and standard deviation for these elements, as well as B, Ca, and Na, measured from the HPHT digestions. ABS filaments contained more elevated concentrations of Al, K, Mg, and Si than the PLA filaments, while Sn was enriched in the PLA filaments compared with ABS. While some elements are known to arise from polymer synthesis methods or color additives, the origin of many of these elements is unclear. The alkali (K, Na) and alkaline (Ca, Mg) metals are likely related to precursor salts or metal salts used as coagulants to recover feedstocks and probing the exact speciation of these materials would not be an easy task [38, 39]. Al and Mg (hydr)oxides, B and Si based materials are all used for flame retardancy and also impart opacity or other material properties, which could explain the differences between manufacturers in the abundances of these elements [40–43]. It has been reported that ABS-silica composites have greater tensile strength, thermal stability, and homogenous morphology, and ABS formulations designed to take advantage of these properties may be the reason for the abundance of Si [36]. Between manufacturers of the same thermoplastic polymer (referred to as Man. 1, 2, and 3 in order to avoid identifying the companies by name), concentrations of a specific element often vary by an order of magnitude.

**Fig. 2:**
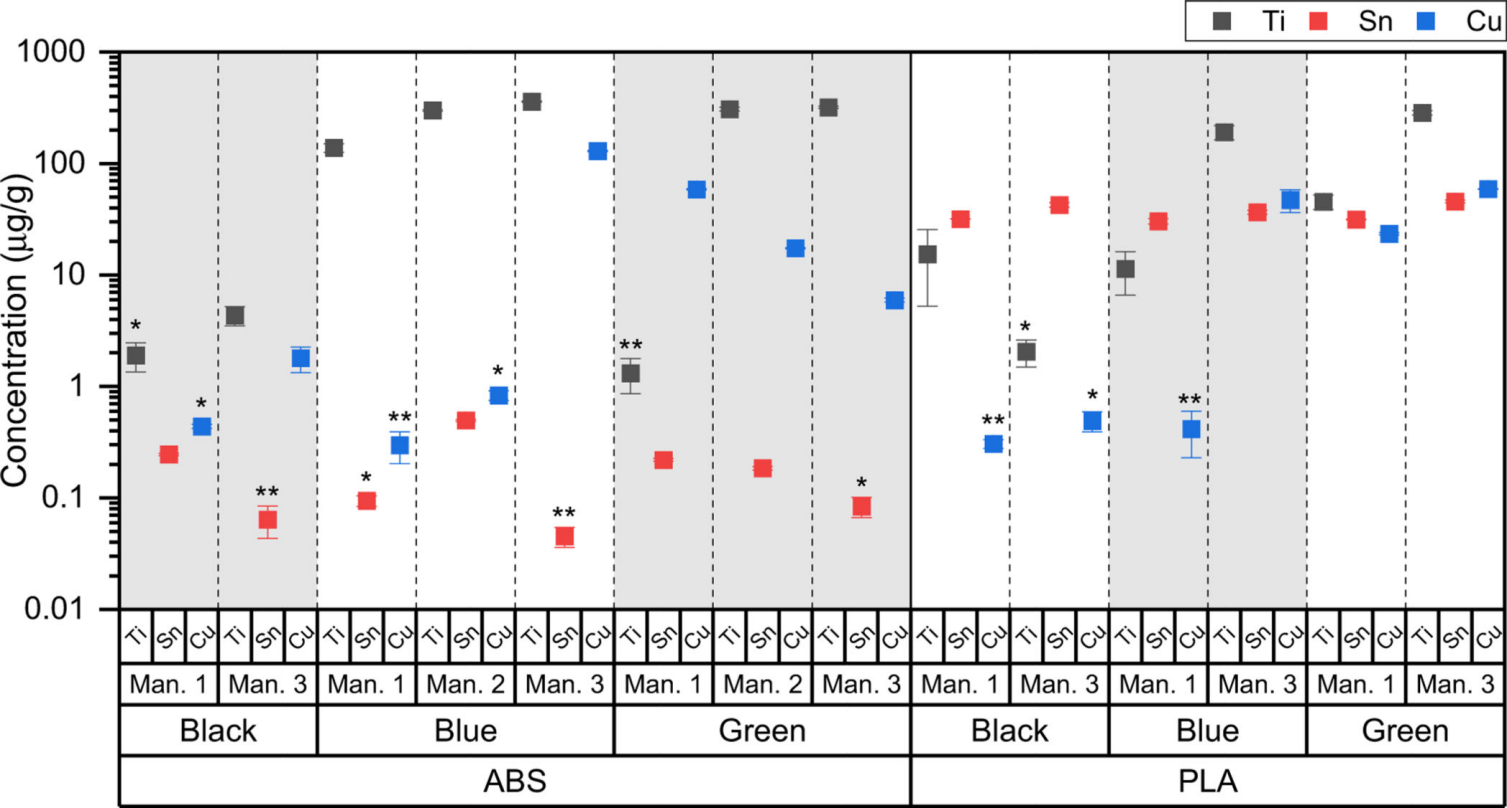
Mean concentrations of Ti, Sn, and Cu (black, red, and blue, respectively) in ABS and PLA filaments, grouped primarily by color and secondarily by manufacturer with error bars representing the standard error. Data below detection and quantification are included, but marked with asterisks (*below MRL, **below MDL)

A closer examination of several commonly abundant elements (Cu, Ti, and Sn) is provided in Fig. 2 for comparisons between manufacturers of ABS and PLA filaments. Variation in metal concentrations between colors (discussed in the next section) was expected due to the role of metal additives as coloring agents, but occasionally filaments that were the same material and color but sourced from differing manufacturers were very different compositionally. The manufacturers are ordered by price point with Man. 1 being the least expensive and Man. 3 being the most expensive. In general, cost does not appear to be a primary factor related to compositional differences, though Al, Si Ti, and Sn in Man. 1 were consistently lower than the equivalent Man. 2 or Man. 3 filaments, suggesting that cost could play a role in the quantity of these elements in the filament. In green ABS filaments, a three-fold higher concentration of Cu was found in Man. 1 as compared with Man. 2. Among all the blue filaments (for both ABS and PLA), Cu concentrations in Man. 3 filaments were over an order of magnitude greater than in Man. 1 or 2. Ti was more than two orders of magnitude more concentrated in Man. 2 and 3 than Man. 1 in the green ABS filaments and no colors of PLA contained the same amount of Ti between manufacturers. While Sn concentrations were similar across all PLA filaments of every color and manufacturer, a variation was observed between different manufacturers of black and blue ABS. There were no consistent trends. These differences between manufacturer sources could likely arise from differences in the synthesis method or formulations used to achieve a particular color.

Sn is prevalent in PLA but not ABS because tin(II) octoate is the main catalyst used in the polymerization of PLA [44, 45]. XAS analysis of the Sn K-edge in these filaments was performed and fitted using standards taken from Impellitteri et al. for Sn speciation in polyvinyl chloride [46]. Sn(II) octoate was not included in analysis due to facility and time restrictions. The full fitting results are provided in the SI along with the spectra and their best fits (Table S7; Fig. S2), but some differences were notable. All six PLA filaments were able to be fit with only two standards, one of which was always tin(IV) oxide (SnO_2_). Man. 1 filaments had fit percentages of 38% – 66% SnO_2_ and Man. 3 filaments had percentages of 66% – 74% S nO_2_. For each, the balance was fit with 29% – 64% of either methyltin trichloride, dibutyltin dichloride, or triphenyltin chloride but there is no pattern for which compound was included in the fit. Overall, this analysis suggests that SnO_2_ may be a prevalent Sn species in PLA filaments that should be considered in health exposure scenarios.

### 3.3 Comparison of the colors

**Fig. 3:**
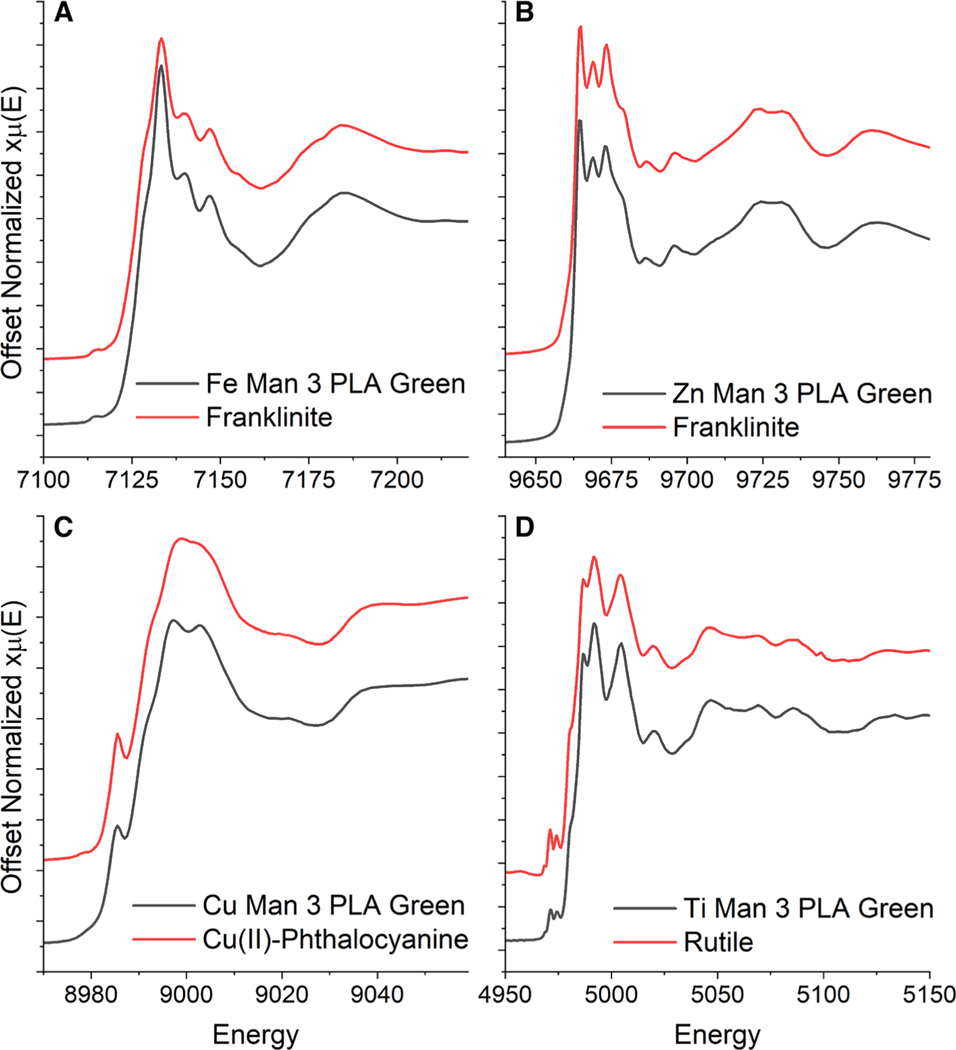
X-ray absorption spectra of **a** iron, **b** zinc, **c** copper, and **d** titanium from the PLA Green filament from Man. 3. Each filament spectrum is the lower black line. The upper red line corresponds to the predominant species (> 90%) identified from the spectra. Included as the upper red line in **a** is franklinite/zinc ferrite, in **b** franklinite/zinc ferrite, in **c** copper(II) phthalocyanine [blue], and in **d** rutile/titanium(IV) oxide

Color appears to be an important driver of compositional differences between filaments of the same type of thermoplastic. For example, Al, K, Na, and Si were significantly higher in Man. 2 ABS blue compared to Man. 2 ABS green (*p*-value < 0.001 for all 4 elements), and in Man. 3 ABS lime green compared to Man. 3 ABS blue or black (*p*-value < 0.001 for all 4 elements; Table 3), which suggests that compositional differences may result from differences in color additives. Black filaments contained the lowest concentrations of Cu, Fe, Ti, and Zn (Table 3) compared to the other colors. The black filaments likely use carbon black as a coloring agent considering its high stability and ease of production [47]. Ti is present in high concentrations, at > 130 μg/g, in 7 of the 10 blue and green filaments. Although Ti can be paired with other elements, such as Ni, Co, or Cr, for colored inorganic pigments [48], it was very likely that these contained titanium dioxide (TiO_2_), which is widely used for its non-toxicity, UV-resistance, and opacifying properties [33]. XAS analysis of filaments with elevated levels (> 50 μg/g) of Ti from Man. 1, 2, and 3 revealed that rutile TiO_2_ was the only detectable phase present (Figs. 3d, S3). The identification of rutile as the species of Ti present in the filaments provides better insight as to why the HPHT method was required for recovery of the Ti present in the filaments.

XAS speciation was also used to identify the primary colorants in the filaments containing Cu, Fe, and Zn (Fig. 3). Only the green PLA filament from Man. 3 had elevated levels of Zn and Fe and both elements had very distinct absorption spectra (Fig. 3). For both elements, this is identifiable as primarily zinc ferrite (ZnFe_2_O_4_), which was fit 98 ± 1% to the Zn spectrum and 87 ± 6% to the Fe spectrum, balanced by 13 ± 6% iron(III) oxide-hydroxide. Zinc ferrite is a yellow–brown pigment that also contributes improved heat stability [48]. Man. 3 PLA green also included Cu, which was fit by 96 ± 1% copper(II) phthalocyanine [blue] (spectra initially provided by Chaboy et al. [49]). Copper(II) phthalocyanine is very widely commercially used as blue (where the phthalocyanine ring is fully hydrogenated) and green to green-yellow (where 14–16 of the hydrogen have been substituted by Cl or Br) pigments because of its economy, heat stability, and chemical resistance [50]. The Ti, Cu, Fe, and Zn spectra of the Man. 3 PLA green filament with each associated primary standard are provided in Fig. 3.

Cu fitting results for the other blue and green filaments also identify copper(II) phthalocyanine as the primary Cu species (Table S6; Fig. S4). Of particular note in the Cu, Fe, and Zn concentration results is that Man. 1 ABS and PLA dark blue and Man. 2 ABS blue do not have appreciable amounts of any of these colorants. This could indicate that these two manufacturers use an organic colorant rather than an inorganic one to achieve their specific shades of blue.

### 3.4 Estimation of aerosol emissions during printing

Previous research that has focused on characterizing emissions from FFF printers indicate that the 3D printing process produces volatile organic compounds and aerosols comprised of ultra-fine particles. In a meta-analysis of published data, Byrley et al. [51] calculated the mean and standard deviation of particle count diameter (PCD, nm) and the range, mean, and standard deviation of particle number emissions rate (PNER, counts/min) for ABS and PLA filaments across sixteen criteria-meeting papers. Using data from Byrley et al. (PCD and PNER), the densities of ABS and PLA (1.05 and 1.24 g/cm^3^, respectively), and making the assumption that the that the elemental composition and concentration from the filament (determined by digestion here) is equivalently partitioned into the aerosols, the theoretical mass of each element present within the aerosol was calculated as a function of emission rate (Table 4).

The estimated exposure to metals or metal oxides is far greater for ABS filaments compared to the PLA (Table 4). This is due to the increased quantity of aerosol produced by ABS filament during the printing process. For ABS filaments, Si and Ti represent the highest concentrations of elements that might be present in the aerosol phase. The calculated emission rates for six elements were compared with the National Institute of Occupational Health and Safety (NIOSH) Recommended Exposure Limits (REL) per 8-h workday, based on a 40-h work week exposure. While the final aerosol concentration is dependent on a variety of factors including room size, ventilation, and air exchange rates, it is plausible to see how concentrations could exceed the REL for Ti, Si, or possibly Cu depending on the filament. Importantly, Byrley et al. reported ranges in their analysis over two orders of magnitude in the PNER reported, which would result in orders of magnitude differences in the concentrations of the metals in the aerosols. Chemical digestion and speciation data demonstrate important differences in filament composition based on color and manufacturer. The wide range of colorants and dyes used by different manufacturers with varying chemical compositions will make it difficult to determine if the reported health effects associated with exposure to aerosols generated during 3D printing are a function of the inorganic chemical composition of the aerosols.

## 4 Conclusion

The study presented here reports on a comparison of several acid-assisted digestion methods in their use for determining the inorganic content of common 3D printing filaments of different polymer types, manufacturers, colors, and specialty applications. Our research indicates that maximum recovery for most elements in PLA filaments was achieved by the use of microwave promoted digestion (M, HT, and HPHT) except for when Ti was present. In those filaments, the HPHT method was required for extraction of the Ti present, which was identified as rutile TiO_2_ by XAS (Fig. S3). Maximum recovery for many of the elements in addition to Ti required the HPHT digestion method for the ABS filaments, and there was a significantly lower recovery when using the M method. Of the inorganic elements that were identified, nearly all of them could be tied to either production processes or desired properties of the filaments. Cu, Fe, and Zn were the primary colorants in the green and blue filaments, identified through XAS as Cu(II) phthalocyanine and zinc ferrite. TiO_2_ was present in many filaments probably as an opacifier or for UV resistance. SnO_2_ was identified in all PLA filaments as a likely byproduct from the catalytic polymerization process that forms PLA.

As estimated from the elemental results determined here and the emission rates for aerosols that could be produced printing with these filaments, our predictions suggest that aerosol concentrations exceeding the NIOSH REL may result. An additional hazard may be posed by environmentally persistent free radicals, a unique class of particulate-based hazard caused by the combustion of organic matter with transition metals or metal oxides present [53–55]. Considering the concentrations and species identified in this study, additional information is required to fully understand the potential human health impacts related to the operation of 3D printers.

The filaments studied in this manuscript are only a very small portion of the 3D printing market and there are many more factors that can be analyzed and assessed. Colors such as orange or red could include greater concentrations of Fe and Cr. Filaments with specialty additives like nanomaterials, flame retardants, or even organic products like beer and coffee, could unintentionally include additional inorganic elements from their own production processes. Metal infused filaments, such as copper and stainless steel, will undoubtedly contain especially high quantities of metal which may pose hazards unique from the more common plastic materials. And more resistant plastics such as polycarbonate or polyethylene terephthalate glycol might even require adjustments to the HPHT method used here. This research presents a necessary assessment of the base materials being used increasingly in 3D printing throughout the world.

## Supplementary Material

Supplementary Information

## Figures and Tables

**Table 1:** Results from the ANOVA and Tukey Means comparison analysis of the elements that were present at quantifiable concentrations, in at least 50% of the filaments tested for two of the digestion methods

Polymer	Element	Max	M	HT	HPHT	HB	*p* value
ABS	Mg	HPHT	b	b	a	a	< 0.001
	Al	M	a	a	a	a	0.510
	Si	HPHT	b	ab	a	b	0.006
	P	HPHT	b	a	a	a	< 0.001
	K	HB	a	a	a	a	0.447
	Ca	HPHT	b	a	a	a	0.006
	Ti	HPHT	b	b	**a**	b	< 0.001
	Cu	HB	b	b	ab	a	0.002
PLA	Ca	HPHT	a	a	a	b	0.028
	Ti	HPHT	b	b	a	b	< 0.001
	V	M	a	a	a	a	0.570
	Cu	HB	a	a	a	a	0.064
	Sn	HB	a	a	a	a	0.211

**Table 2:** Average concentrations (in μg/g) of select elements from 3 to 5 replicates of HPHT digestion of colored ABS and PLA filaments

ABS filaments	Al	B	Ca	K	Mg	Na	Si	Sn
Man. 1 average		***1.58* ± *1.14***	**258 ± 20**	**20.3 ± 7.6**		***44.6* ± *6.2***	**56.7 ± 7.7**	**0.187 ± 0.071**
Man. 1 black			271 ± 17	22.5 ± 4.2		*49.0* ± *5.6*	48.0 ± 5.5	0.246 ± 0.01
Man. 1 dark blue			269 ± 9	11.8 ± 0.5		*40.2* ± *18.8*	59.6 ± 11.0	*0.094* ± *0.018*
Man. 1 grass green		*1.58* ± *1.14*	*236* ± *8*	26.6 ± 3.9			62.6 ± 1.9	0.220 ± 0.011
Man. 2 average	**587 ± 668**	***1.49* ± *0.32***	**327 ± 30**	**61.3 ± 57.6**	***44.9* ± *2.8***	**539 ± 558**	**784 ± 843**	**0.341 ± 0.171**
Man. 2 blue	1060 ± 30	*1.49* ± *0.32*	348 ± 8	102 ± 4	*42.9* ± *1.3*	953 ± 23	1390 ± 50	0.497 ± 0.019
Man. 2 green	115 ± 1		306 ± 5	20.5 ± 0.4	*46.9* ± *0.5*	125 ± 2	188 ± 3	0.185 ± 0.012
Man. 3 average	**159 ± 77**	**46.0 ± 40.5**		**35.6 ± 27.2**	**198 ± 12**	**85.3 ± 46.6**	**201 ± 131**	***0.078* ± *0.040***
Man. 3 black		83.5 ± 26.0		22.6 ± 1.7	193 ± 4	*61.1* ± *4.1*	136 ± 3	
Man. 3 blue	104 ± 14	3.06 ± 2.63		17.4 ± 3.1	211 ± 3	*55.8* ± *5.4*	115 ± 3	
Man. 3 lime green	213 ± 14	51.5 ± 26.5		66.9 ± 5.7	189 ± 13	139 ± 7	351 ± 12	*0.078* ± *0.040*

PLA filaments	Al	B	Ca	K	Mg	Na	Si	Sn^a^

Man. 1 average			**371 ± 57**			**85.8 ± 39.9**	***25.2* ± *1.9***	**30.9 ± 0.7**
Man. 1 black			*250* ± *5*			*56.2* ± *2.1*	*23.1* ± *3.1*	31.8 ± 0.1
Man. 1 dark blue			331 ± 31			*57.6* ± *5.1*	*23.8* ± *5.1*	30.4 ± 3.1
Man. 1 green			411 ± 14			114 ± 12	*26.5* ± *4.3*	31.4 ± 0.5
Man. 3 average	***48.3* ± *10.1***	**2.14 ± 1.70**	***154* ± *27***			**88.1 ± 52.3**	***20.9* ± *2.4***	**41.6 ± 4.6**
Man. 3 black						*64.9* ± *2.9*	*19.6* ± *1.6*	42.5 ± 3.5
Man. 3 blue						*51.4* ± *6.3*	*19.4* ± *3.3*	36.6 ± 2.6
Man. 3 green	*48.3* ± *10.1*	2.14 ± 1.70	*154* ± *27*			148 ± 10	*23.6* ± *1.9*	45.7 ± 2.3

Bolded text presents the average concetation of metals present in all of the filaments for a specific manufacturer

Blanks indicate that measured concentration was below detection limits, and italics indicate the measured concentration was below the calculated reporting limits for the HPHT digestion

a XAS linear combination fitting results are provided in Table S7

**Table 3:** Average concentrations (in μg/g) of select elements from 3 to 5 replicates of HPHT digestion of colored ABS and PLA filaments

	Cu	Fe	Ti	Zn
ABS filaments				
Man. 1 black	*0.439* ± *0.032*	6.66 ± 3.99	*1.84* ± *0.88*	
Man. 3 black	1.80 ± 0.93	16.7 ± 3.7	4.35 ± 1.7	
Man. 1 dark blue		*5.84* ± *2.47*	138 ± 21	
Man. 2 blue	*0.833* ± *0.140*	16.4 ± 0.9	300 ± 9	
Man. 3 blue	130 ± 2^a^	9.38 ± 1.21	360 ± 7	*14.4* ± *1.8*
Man. 1 grass green	58.7 ± 1.5^a^			
Man. 2 green	17.4 ± 0.4^a^	*3.99* ± *0.08*	308 ± 21	
Man. 3 lime green	5.94 ± 0.52	22.3 ± 4.7	320 ± 16	*13.0* ± *1.5*
PLA filaments				
Man. 1 black		*2.91* ± *0.10*	15.4 ± 17.6	
Man. 3 black	*0.440* ± *0.200*	*7.43* ± *1.45*	*1.94* ± *0.95*	
Man. 1 dark blue		*4.01* ± *0.21*	11.4 ± 8.3	
Man. 3 blue	47.1 ± 18.8^a^	*4.05* ± *0.41*	191 ± 47.7	
Man. 1 green	23.5 ± 0.8^a^	*4.91* ± *1.3*	45.4 ± 11.7	
Man. 3 green	59.1 ± 0.7^b^	605 ± 39^b^	286 ± 22	299 ± 15^b^

Blanks indicate that an average was not detected, and italics indicate that it was below the MRL

a XAS linear combination fitting results are provided in Table S6 and Figure S1

b XAS linear combination fitting results are provided in Table S6 and Fig. 3

**Table 4:** Calculated rate of elemental composition and concentration in aerosol emissions produced during 3D printing assuming equivalent partitioning from the filament into the aerosol

Material	Manufacturer	Color	Mean PCD*	Mean PNER Low*	Si	Ti	Fe	Cu	Sn	Zn
			nm	counts/min	μg/min					
ABS	Man. 2	Blue	48.5	1.94E+10	4.36	21.96	1.20	0.06	0.00	
ABS	Man. 3	Black	48.5	1.94E+10	9.91	0.32	1.22	0.13	0.00	
ABS	Man. 3	Blue	48.5	1.94E+10	8.43	26.34	0.69	9.48	0.00	1.05
ABS	Man. 3	Green	48.5	1.94E+10	25.70	23.37	1.63	0.43	0.00	0.95
PLA	Man. 1	Green	40.4	2.08E+09	0.14	0.24	0.03	0.13	0.17	
PLA	Man. 3	Black	40.4	2.08E+09	0.10	0.01	0.04	0.00	0.23	
PLA	Man. 3	Blue	40.4	2.08E+09	0.10	1.02	0.02	0.25	0.20	
PLA	Man. 3	Green	40.4	2.08E+09	0.13	1.53	3.23	0.32	0.24	1.60
NIOSH recommended exposure limit (mg/m^3^ per 8 h)**					6000^†^	300	5000	1000	2000	5000

* Values are based on data presented by Byrley et al. [51]

** Values obtained from the NIOSH Pocket Guide to Chemical Hazards [52]

† Amorphous silica (SiO_2_)

## Data Availability

All data presented in the manuscript will be made available for public download at www.data.gov by searching the manuscript title.
