# Supplementary material for: Variability in the inorganic composition of colored acrylonitrile–butadiene–styrene and polylactic acid filaments used in 3D printing
Source: SN Appl Sci. Author manuscript; Available in PMC 2023 Jun 28. (PMC10305001; doi:10.1007/s42452-022-05221-7)
Supplement: Supplementary Information [file NIHMS1868229-supplement-Supplementary_Information.docx]

Supporting Information for:

*Variability in the Inorganic Composition of Colored Acrylonitrile-butadiene-styrene and Polylactic Acid Filaments Used in 3D Printing*

Derek M. Peloquin^ab^, Logan N. Rand^a^, Eric J. Baumann^ac^, Ali Gitipour^ac^, Joanna Matheson^d^, Todd P. Luxton^a*^

^a^U.S. Environmental Protection Agency, Office of Research and Development, Center for Environmental Research and Emergency Response, Cincinnati, OH, USA

^b^Current Address: U.S. Food and Drug Administration, Office of Regulatory Affairs, Forensic Chemistry Center, Cincinnati, OH 45237

^c^Current Address: U.S. Environmental Protection Agency, Office of Research and Development, Center for Environmental Measurements and Modeling, Research Triangle Park, NC, USA

dU.S. Consumer Product Safety Commission, Office of Hazard Identification and Reduction, Bethesda, MD, USA.

*Todd Luxton,

5995 Center Hill Ave,

Cincinnati, OH 45224 USA

[luxton.todd@epa.gov](mailto:luxton.todd@epa.gov)

**Supporting Information**

9 Pages, 7 Tables, 3 Figures

**Table S1.** Instrument and method detection and reporting limits (ppb) for the masses, elements, and modes used^1^.

|  | | | Instrument Limits | | Method M | | Method HT | | Method HPHT | | Method HB | |
| --- | --- | --- | --- | --- | --- | --- | --- | --- | --- | --- | --- | --- |
| Mass | Element | Mode | IDL | IRL | MDL | MRL | MDL | MRL | MDL | MRL | MDL | MRL |
| 27 | Al, Aluminum | He | 0.376 | 1.127 | 57.234 | 171.701 | 30.679 | 92.037 | 59.026 | 177.079 | 56.387 | 169.162 |
| 75 | As, Arsenic^2^ | He | 0.021 | 0.064 | 0.067 | 0.202 | 0.025 | 0.074 | 0.054 | 0.163 | 0.047 | 0.142 |
| 11 | B, Boron | He | 0.785 | 2.356 | 1.698 | 5.093 | 1.120 | 3.360 | 0.785 | 2.356 | 1.397 | 4.190 |
| 137 | Ba, Barium | He | 0.236 | 0.709 | 0.236 | 0.709 | 0.236 | 0.709 | 0.622 | 1.865 | 0.236 | 0.709 |
| 9 | Be, Beryllium | no gas | 0.008 | 0.023 | 0.008 | 0.023 | 0.008 | 0.023 | 0.008 | 0.023 | 0.072 | 0.217 |
| 44 | Ca, Calcium | no gas | 1.110 | 3.329 | 66.947 | 200.842 | 24.441 | 73.324 | 157.442 | 472.327 | 26.496 | 79.488 |
| 111 | Cd, Cadmium^2^ | no gas | 0.084 | 0.251 | 0.145 | 0.436 | 0.084 | 0.251 | 0.084 | 0.251 | 0.084 | 0.251 |
| 59 | Co, Cobalt | He | 0.002 | 0.007 | 0.088 | 0.265 | 0.136 | 0.407 | 0.194 | 0.581 | 0.215 | 0.646 |
| 52 | Cr, Chromium | He | 0.014 | 0.043 | 0.847 | 2.542 | 0.358 | 1.073 | 2.920 | 8.760 | 1.988 | 5.963 |
| 63 | Cu, Copper | no gas | 0.012 | 0.036 | 1.363 | 4.088 | 0.804 | 2.412 | 0.693 | 2.080 | 2.557 | 7.672 |
| 56 | Fe, Iron | He | 0.095 | 0.285 | 6.632 | 19.896 | 8.692 | 26.076 | 6.551 | 19.652 | 51.232 | 153.695 |
| 39 | K, Potassium | no gas | 2.012 | 6.036 | 2.012 | 6.036 | 2.012 | 6.036 | 2.012 | 6.036 | 2.012 | 6.036 |
| 24 | Mg, Magnesium | He | 0.194 | 0.583 | 15.798 | 47.394 | 2.397 | 7.190 | 42.747 | 128.241 | 18.503 | 55.508 |
| 55 | Mn, Manganese | He | 0.017 | 0.052 | 0.267 | 0.800 | 0.117 | 0.350 | 0.728 | 2.183 | 0.796 | 2.389 |
| 95 | Mo, Molybdenum | He | 0.009 | 0.028 | 0.028 | 0.084 | 0.037 | 0.111 | 0.759 | 2.276 | 0.836 | 2.509 |
| 23 | Na, Sodium | He | 0.485 | 1.455 | 64.798 | 194.394 | 38.091 | 114.273 | 76.588 | 229.763 | 46.679 | 140.036 |
| 60 | Ni, Nickel | He | 0.031 | 0.093 | 0.735 | 2.206 | 0.526 | 1.579 | 0.751 | 2.254 | 1.600 | 4.801 |
| 31 | P, Phosphorus | no gas | 2.373 | 7.118 | 37.218 | 111.655 | 40.603 | 121.809 | 39.454 | 118.361 | 119.700 | 359.099 |
| 208 | Pb, Lead^2^ | no gas | 0.011 | 0.034 | 0.474 | 1.422 | 0.224 | 0.671 | 2.135 | 6.406 | 1.235 | 3.706 |
| 121 | Sb, Antimony | He | 0.012 | 0.037 | 0.514 | 1.543 | 0.477 | 1.431 | 0.546 | 1.638 | 0.733 | 2.198 |
| 78 | Se, Selenium | He | 0.539 | 1.616 | 0.539 | 1.616 | 0.667 | 2.002 | 0.539 | 1.616 | 0.772 | 2.317 |
| 28 | Si, Silicon | no gas | 1.003 | 3.008 | 32.358 | 97.073 | 64.031 | 192.092 | 26.951 | 80.853 | 64.105 | 192.314 |
| 118 | Sn, Tin | He | 0.007 | 0.022 | 0.342 | 1.025 | 0.624 | 1.873 | 0.113 | 0.340 | 0.949 | 2.848 |
| 88 | Sr, Strontium | He | 0.014 | 0.043 | 0.514 | 1.542 | 0.036 | 0.109 | 1.027 | 3.081 | 1.289 | 3.867 |
| 47 | Ti, Titanium | He | 0.039 | 0.117 | 1.322 | 3.965 | 1.063 | 3.189 | 2.447 | 7.342 | 1.058 | 3.175 |
| 205 | Tl, Thallium | He | 0.015 | 0.045 | 0.015 | 0.045 | 0.015 | 0.045 | 0.015 | 0.045 | 0.608 | 1.825 |
| 51 | V, Vanadium^2^ | He | 0.018 | 0.053 | 0.087 | 0.260 | 0.018 | 0.053 | 0.018 | 0.053 | 0.018 | 0.053 |
| 66 | Zn, Zinc | He | 0.216 | 0.649 | 14.497 | 43.492 | 5.754 | 17.263 | 28.146 | 84.439 | 17.198 | 51.595 |
| ^1^Internal standard elements used were scandium-45, germanium-72, yttrium-89, indium-115^2^, terbium-159, and bismuth-209.  ^2^Correction equations were applied to vanadium-51, arsenic-75, cadmium-111, indium-115, and lead-208 according to EPA Method 6020B. | | | | | | | | | | | | |

**Table S2**. Summary of elements measured in the ABS filaments used in the current study and which met the criteria for inclusion in the statistical comparison of digestion methods. “D” indicates that the average of 3 to 5 measurements was above the method detection limit, but less than the method reporting limit, whereas “Q” indicates the average concentration was above the method reporting limit (see Table S1). The green highlighted cells at the bottom of the table indicate if an element was present in >50% of the filaments analyzed for a specific digestion method.

**Table S3.** Summary of elements measured in the PLA filaments used in the current study and which met the criteria for inclusion in the statistical comparison of digestion methods. “D” indicates that the average of 3 to 5 measurements was above the method detection limit, but less than the method reporting limit, whereas “Q” indicates the average concentration was above the method reporting limit (see Table S1). The green highlighted cells at the bottom of the table indicate if an element was present in >50% of the filaments analyzed for a specific digestion method.

**Table S4**. Mean concentrations in ABS filaments and statistical comparison results from the ANOVA and Tukey Test of the elements that were present at quantifiable concentrations, in at least 50% of the filaments tested for two of the digestion methods. SE is the Standard Error and in the Comparison column, statistically significant differences between digestion methods are denoted with lowercase letters, such that “a” and “b” results are different from each other, “a” results are not significantly different from each other, “b” results are not significantly different from each other, and “ab” results are not significantly different from “a” or “b” results.

| Polymer | Element | Digestion | Concentration (μg/g) | | Comparison |
| --- | --- | --- | --- | --- | --- |
|  |  |  | Mean | SE |  |
| ABS | Mg | M | 16.14 | 2.97 | b |
| ABS | Mg | HT | 33.90 | 7.48 | b |
| ABS | Mg | HPHT | 145.58 | 17.43 | a |
| ABS | Mg | HB | 113.79 | 13.22 | a |
| ABS | Al | M | 353.67 | 159.68 | a |
| ABS | Al | HT | 196.47 | 56.41 | a |
| ABS | Al | HPHT | 351.21 | 104.24 | a |
| ABS | Al | HB | 298.20 | 82.75 | a |
| ABS | Si | M | 34.88 | 5.25 | b |
| ABS | Si | HT | 147.51 | 36.36 | ab |
| ABS | Si | HPHT | 292.19 | 78.82 | a |
| ABS | Si | HB | 116.19 | 12.87 | b |
| ABS | P | M | 29.71 | 3.79 | b |
| ABS | P | HT | 74.65 | 4.18 | a |
| ABS | P | HPHT | 82.06 | 5.77 | a |
| ABS | P | HB | 77.57 | 1.84 | a |
| ABS | K | M | 27.23 | 11.13 | a |
| ABS | K | HT | 28.20 | 5.32 | a |
| ABS | K | HPHT | 38.04 | 5.72 | a |
| ABS | K | HB | 42.91 | 12.21 | a |
| ABS | Ca | M | 129.44 | 24.74 | b |
| ABS | Ca | HT | 254.77 | 20.46 | a |
| ABS | Ca | HPHT | 286.12 | 10.43 | a |
| ABS | Ca | HB | 245.88 | 27.20 | a |
| ABS | Ti | M | 1.30 | 0.21 | b |
| ABS | Ti | HT | 21.06 | 10.35 | b |
| ABS | Ti | HPHT | 206.00 | 30.06 | a |
| ABS | Ti | HB | 3.42 | 0.43 | b |
| ABS | Cu | M | 4.84 | 1.26 | b |
| ABS | Cu | HT | 19.86 | 4.00 | b |
| ABS | Cu | HPHT | 27.42 | 8.92 | ab |
| ABS | Cu | HB | 52.48 | 13.46 | a |

**Table S5**. Mean concentrations in PLA filaments and statistical comparison results from the ANOVA and Tukey Test of the elements that were present at quantifiable concentrations, in at least 50% of the filaments tested for two of the digestion methods. SE is the Standard Error and in the Comparison column, statistically significant differences between digestion methods are denoted with lowercase letters, such that “a” and “b” results are different from each other, “a” results are not significantly different from each other, “b” results are not significantly different from each other, and “ab” results are not significantly different from “a” or “b” results.

| Polymer | Element | Digestion | Concentration (μg/g) | | Comparison |
| --- | --- | --- | --- | --- | --- |
|  |  |  | Mean | SE |  |
| PLA | Ca | M | 308.17 | 18.12 | a |
| PLA | Ca | HT | 244.29 | 29.92 | a |
| PLA | Ca | HPHT | 286.51 | 29.19 | a |
| PLA | Ca | HB | 183.50 | 27.08 | a |
| PLA | Ti | M | 2.74 | 0.81 | b |
| PLA | Ti | HT | 4.53 | 1.04 | b |
| PLA | Ti | HPHT | 91.81 | 26.59 | a |
| PLA | Ti | HB | 5.67 | 0.68 | b |
| PLA | V | M | 0.25 | 0.04 | a |
| PLA | V | HT | 0.23 | 0.04 | a |
| PLA | V | HPHT | 0.16 | 0.05 | a |
| PLA | V | HB | 0.20 | 0.04 | a |
| PLA | Cu | M | 31.09 | 4.51 | a |
| PLA | Cu | HT | 46.40 | 4.86 | a |
| PLA | Cu | HPHT | 32.56 | 7.18 | a |
| PLA | Cu | HB | 49.31 | 5.43 | a |
| PLA | Sn | M | 33.45 | 1.82 | a |
| PLA | Sn | HT | 36.64 | 1.06 | a |
| PLA | Sn | HPHT | 36.43 | 1.50 | a |
| PLA | Sn | HB | 37.87 | 1.43 | a |

**Table S6.** Linear combination fitting results of copper, iron, and zinc in ABS and PLA filaments using normalized µ(E) and a fitting range of -30 to 100 eV relative to the absorption edge. Although other compounds were included in the LCF analysis, they were omitted on providing no percentage contribution.

| **Copper (Cu)** | CuPc (%) | CuCl_2_ (%) | Cu (%) | Cu(OAc)_2_ (%) | Reduced χ^2^ | R-Factor |
| --- | --- | --- | --- | --- | --- | --- |
| Man. 1 ABS grass green | 95 | 5 |  |  | 0.0001695 | 0.0007444 |
| Man. 2 ABS green | 96 | 3 |  |  | 0.0001629 | 0.0007186 |
| Man. 3 ABS blue | 96 |  | 3 |  | 0.0000906 | 0.0004146 |
| Man. 1 PLA green | 92 |  |  | 8 | 0.0002657 | 0.0014353 |
| Man. 3 PLA blue | 84 |  | 16 |  | 0.0000936 | 0.0004838 |
| Man. 3 PLA green | 96 |  |  | 5 | 0.0001328 | 0.0005976 |
| *CuPc = copper(II) phthalocyanine [blue], CuCl_2_ = copper(II) chloride, Cu = zero valent copper, Cu(OAc)_2_ = copper(II) acetate | | | | | | |
| **Iron (Fe)** | ZnFe_2_O_4_ (%) | | FeO(OH) (%) | | Reduced χ^2^ | R-Factor |
| Man. 3 PLA green | 87 | | 13 | | 0.0014585 | 0.0073983 |
| *ZnFe_2_O_4_ = zinc ferrite, FeO(OH) = iron(III) oxide-hydroxide | | | | | | |
| **Zinc (Zn)** | ZnFe­_2_O_4_ (%) | | ZnO (%) | | Reduced χ^2^ | R-Factor |
| Man. 3 PLA green | 98 | | 1 | | 0.0001254 | 0.0005498 |
| *ZnFe_2_O_4_ = zinc ferrite, ZnO = zinc oxide | | | | | | |


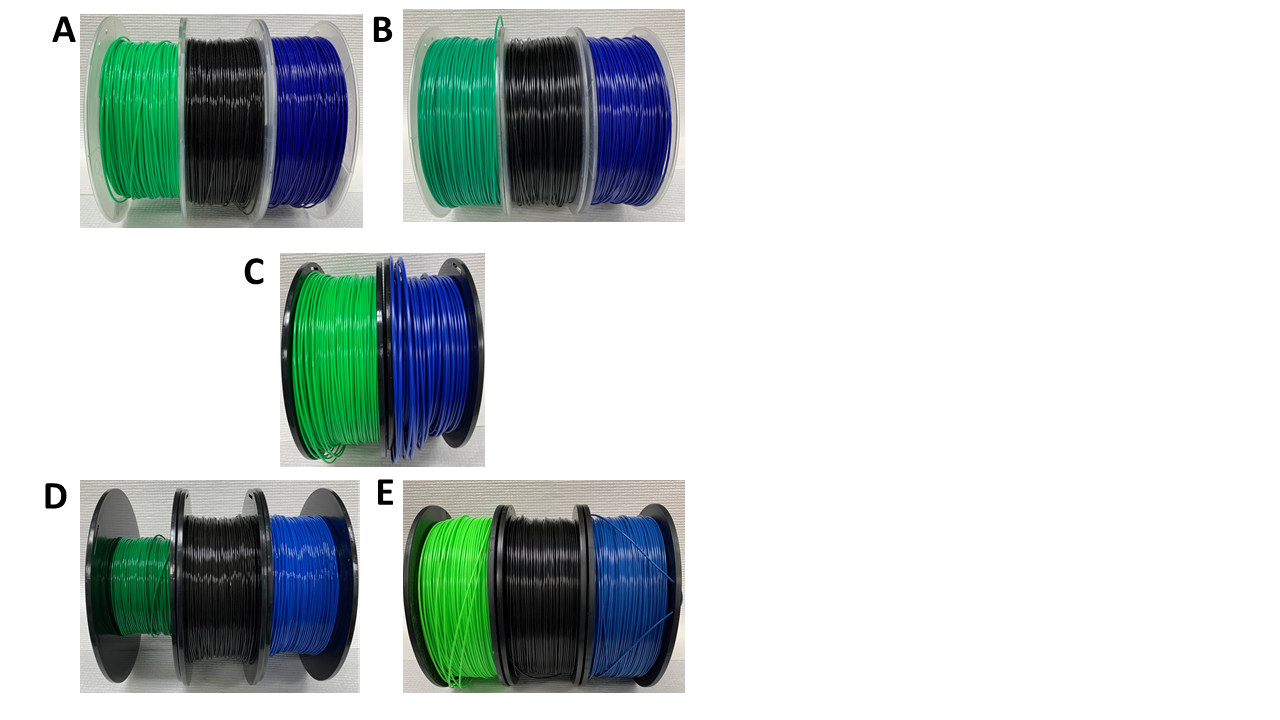


Figure S1. Color images of the thermoplastic filaments used in the current study. A) Manufacturer 1 PLA Filaments, B) Manufacuter 1 ABS Filaments, C) Manufacturer 2 ABS Filaments, D) Manaufacturer 3 PLA filaments, E) Manufacturer 3 ABS Filaments.

**Table S7.** Linear combination fitting results of Sn in PLA filaments using normalized µ(E) and a fitting range of -30 to 100 eV relative to the absorption edge. Although other compounds were included in the LCF analysis, they were omitted on providing no percentage contribution.

| **Tin (Sn)** | SnO_2_ (%) | MeSnCl_3_ (%) | Bu_2_SnCl_2_ (%) | Ph_3­_SnCl (%) | Reduced χ^2^ | R-Factor |  |
| --- | --- | --- | --- | --- | --- | --- | --- |
| Man. 1 black | 38 | 64 |  |  | 0.0001888 | 0.0011424 |  |
| Man. 1 dark blue | 41 |  | 63 |  | 0.0002149 | 0.0011697 |  |
| Man. 1 green | 66 |  |  | 35 | 0.0001028 | 0.0005370 |  |
| Man. 3 black | 74 |  | 29 |  | 0.0002328 | 0.0012134 |  |
| Man. 3 blue | 69 | 34 |  |  | 0.0003321 | 0.0019809 |  |
| Man. 3 green | 66 |  | 36 |  | 0.0000894 | 0.0004924 |  |
| *SnO_2_ = tin(IV) oxide, MeSnCl_3_ = methyltin trichloride, Bu_2_SnCl_2_ = dibutyltin dichloride, Ph_3_SnCl = triphenyltin chloride | | | | | | | |


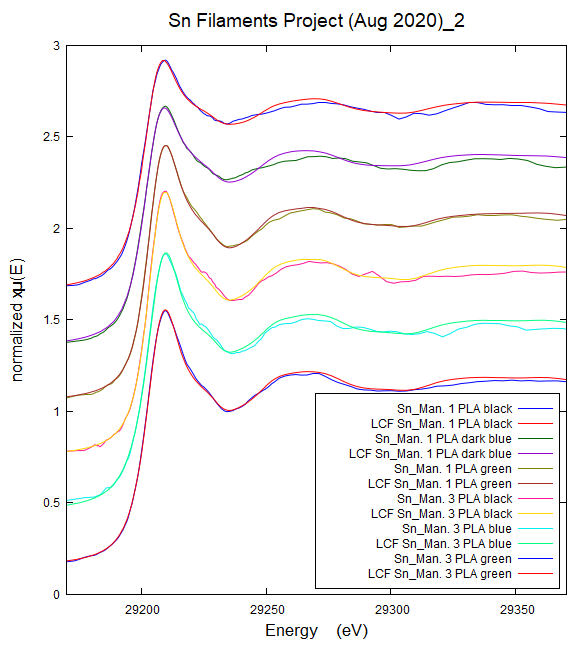


**Figure S2.** Sn K-edge spectra of PLA filaments with lines-of-best-fit from linear combination fitting (LCF) of the standards reported in Table S8.

**
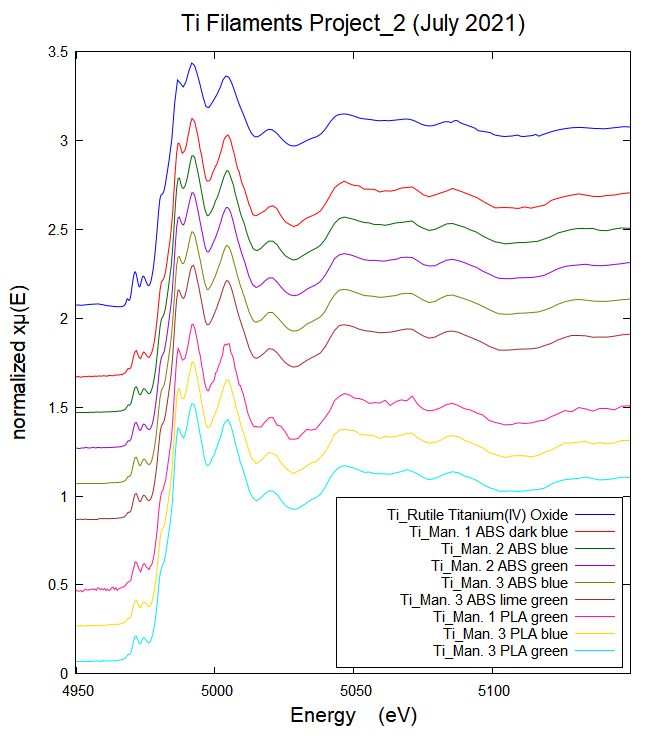
**

**Figure S3.** Ti K-edge spectra of rutile titanium(IV) oxide (trace 1), ABS filaments (traces 2–6), and PLA filaments (traces 7–9). No other standards assessed (anatase titanium(IV) oxide, brookite titanium(IV) oxide, titanium(III) oxide) provided any contribution with LCF.


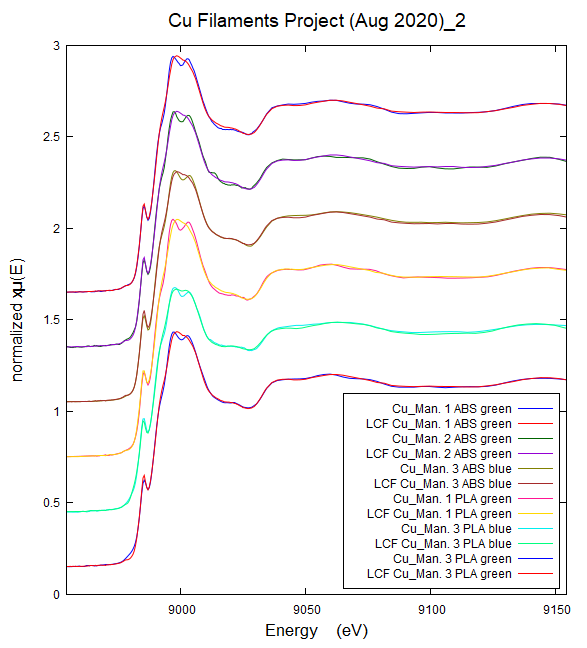


**Figure S*4*.** Cu K-edge spectra of ABS and PLA filaments with lines-of-best-fit from linear combination fitting (LCF) of the standards reported in Table S6.
